# Serum Cystatin C Trajectory Is a Marker Associated With Diabetic Kidney Disease

**DOI:** 10.3389/fendo.2022.824279

**Published:** 2022-05-11

**Authors:** Nana Wang, Zhenyu Lu, Wei Zhang, Yu Bai, Dongmei Pei, Ling Li

**Affiliations:** ^1^ Endocrinology Department, Shengjing Hospital of China Medical University, Shenyang, China; ^2^ Department of Health Management, Shengjing Hospital of China Medical University, Shenyang, China

**Keywords:** cystatin C, diabetic kidney disease, latent class growth mixed modeling, trajectory, velocity

## Abstract

**Objective:**

To explore the association of the trajectory of serum Cystatin C (Cysc) with diabetic kidney disease (DKD), a retrospective cohort study of Chinese subjects was carried out.

**Method:**

A review of 2,928 diabetes mellitus (DM) patients admitted to the clinic and ward of the Endocrinology Department, Shengjing Hospital of China Medical University from January 1, 2014 to December 31, 2014 was performed. Subsequent visits to the hospital were followed until December 31, 2020. The primary endpoint was the incidence of DKD as diagnosed by urinary albumin/creatinine ratio ≥30 mg/g and/or estimated glomerular filtration rate <60 ml/min per 1.73 m^2^. Healthy control subjects were identified from a health checkup database in Shengjing Hospital from 2016 to 2019. The latent class growth mixed modeling (LCGMM) method was used to analyze latent classes of serum Cysc in healthy and DM subjects. Finally, the hazard ratios (HRs) of latent classes of Cysc in DM subjects were analyzed by Cox regression analysis.

**Results:**

A total of 805 type 2 diabetes mellitus (T2DM) and 349 healthy subjects were included in the trial. The HRs of quartiles of baseline Cysc in T2DM subjects were 7.15 [95% confidence interval (CI), 2.79 to 25.57], 2.30 (95% CI, 1.25 to 4.24), and 2.05 (95% CI, 1.14 to 3.70), respectively, for quartile 4 (Q4), Q3, and Q2 when compared with Q1. Through LCGMM, a 1-class linear model was selected for the Cysc latent class in healthy subjects. In contrast, a 3-class linear model was selected for that in DM subjects. The slopes of the three latent classes in T2DM subjects were larger than the slope in healthy subjects. The HRs of incident DKD were 3.43 (95% CI, 1.93 to 6.11) for the high-increasing class and 1.80 (95% CI, 1.17 to 2.77) for the middle-increasing class after adjusting for confounding variables.

**Conclusions:**

Patients with T2DM had a higher velocity of increase in Cysc than healthy subjects. Patients with high baseline Cysc values and high latent increasing velocity of Cysc had a higher risk of developing DKD in later life. More attention should be paid to patients with these high-risk factors.

## Introduction

Diabetic kidney disease (DKD) is one of the major chronic microvascular complications in diabetes mellitus (DM) and a main cause of end-stage renal disease (ESRD). It accounts for almost half of all incident cases of ESRD in DKD patients (1). Chronic kidney disease caused by DM is defined by a persistent estimated glomerular filtration rate (eGFR) of <60 ml/min per 1.73 m^2^ or a urinary albumin/creatinine ratio (UACR) of >30 mg/g for more than 3 months (2). Repeated assessment of UACR in two to three samples together with the eGFR is regarded as the best standard screening for DKD (3). However, recent studies show that DKD can occur without increased albuminuria. The Diabetes Control and Complications Trial (DCCT)/Epidemiology of Diabetes Interventions and Complications (EDIC) study demonstrated that 24% of new-onset DKD patients with type 1 diabetes mellitus (T1DM) progressed to eGFR < 60 ml/min per 1.73 m^2^ but had albumin excretion rates < 30 mg/24 h at all prior evaluations (4). This indicates that the current standard screening of albuminuria may miss some DKD patients. Moreover, in most cases, decreased eGFR is not an earlier biomarker than proteinuria in the early diagnosis of DKD. Therefore, neither albuminuria nor eGFR is an early sensitive marker of DKD. Because of the severity and heavy burden of DKD, early diagnosis is a crucial topic in the prevention and treatment of DM. Therefore, a new biomarker for early diagnosis of DKD is necessary.

Cystatin C (Cysc) is a low-molecular-weight protein (13 kDa) (5) and is a member of the cystatin superfamily of cysteine proteinase inhibitors. It is produced by all nucleated cells at a constant rate (6). Recently, several studies showed that serum Cysc is a better marker of declining GFR in DM patients than serum creatinine (Scr) (7). Most of the evidence came from cross-sectional studies demonstrating that serum Cysc levels in DKD patients were significantly higher when compared to those in DM cases without DKD (8–10). The elevation of Cysc was correlated with decreased GFR and elevated UACR (11, 12), and serum Cysc performed better compared with Scr and albuminuria in detecting mild diabetic nephropathy (13, 14). In addition, equations using Cysc to calculate eGFR were better than equations using creatinine at predicting the mild stage of chronic kidney disease for DM patients (15–17), indicating that Cysc may be a potential biomarker for early diagnosis of DKD (18). Limited short-term longitudinal analysis demonstrated that Cys-eGFR equations predict GFR changes better in 2 years (19), and serum Cysc correlates with renal function decline in T1DM in 1 year (20). Meanwhile, some investigations held opposite views. A study by Iliadis et al. in 488 type 2 diabetes mellitus (T2DM) patients in Greece showed that eGFRcys did not provide better GFR estimation than eGFRcre (21). Another study by Oddoze et al. showed that serum Cysc is not better than Scr for estimating GFR in patients with steady-state diabetes using ROC curves (22). Nevertheless, Cysc is a promising new biomarker for diagnosis of DKD. Until now, large, long-term longitudinal studies monitoring Cysc in the incidence of DKD have not been performed. In particular, the dynamic changes of Cysc in healthy and DM subjects throughout their lifetime are overlooked.

In this study, we included 805 T2DM subjects without DKD at baseline, and followed them for 6 years, analyzing the trajectory of Cysc increase and its association with the incidence of DKD. We also compared the velocity of increase of Cysc in healthy and T2DM subjects in their lifetime. To our knowledge, this is the first investigation to compare the latent trajectory of serum Cysc in healthy and T2DM subjects by latent class growth mixed modeling (LCGMM) with a long follow-up, and provide a new insight into the association of Cysc trajectory with an incidence of DKD.

## Materials and Methods

### Subjects

We reviewed 2,928 DM patients admitted to the clinic and ward of the Endocrinology Department, Shengjing Hospital of China Medical University from January 1, 2014 to December 31, 2014. Subsequent visits to the clinic and ward in the hospital were followed until December 31, 2020. All the patients came from four provinces of China, including Liaoning, Jilin, Heilongjiang, and Neimenggu.

Subjects with T2DM, aged 18–70 years, were included in the study. The exclusion criteria were as follows: T1DM, baseline UACR ≥30 mg/g, baseline eGFR <60 ml/min per 1.73 m^2^, a diagnosis of DKD in the hospital information system (HIS) record at baseline, severe liver dysfunction, history of malignancy, hyperthyroidism, hypothyroidism, pregnancy, missing data for UACR, Cysc or Scr, less than 3 visits, or missing endpoint data in 2019–2020. Subjects’ data after diagnosis of DKD were excluded from the analyses.

Healthy subjects were collected from a health checkup database in Shengjing Hospital. In this project, subjects received health checkups every year from 2016 to 2019. We collected Cysc data of subjects from 2016 to 2019 and excluded subjects with diagnosed or new-onset pre-diabetes, DM, hypertension, thyroid disease, malignancy, pregnancy, other diagnosed diseases, or less than 3 visits.

All studies were approved by the Ethical Review Committee of Shengjing Hospital of China Medical University (No. 2019PS089J for health checkup trial and No. 2021PS755K for DKD trial) and conducted in accordance with the guidelines of the Declaration of Helsinki. Written informed consent was obtained from each participant.

### Data Collection

Data were collected for age, height, systolic and diastolic blood pressure (SBp and DBp, respectively), and biochemical indices including serum Cysc, UACR, HbA_1c_, fasting plasma glucose (FPG), serum lipid, liver function, and kidney function. Present history, previous history, personal history, and family history were collected from the HIS.

Serum Cysc was measured by latex-enhanced immunoturbidimetric assay (Beijing Strong Biotechnologies, Inc., Beijing, China). Serum and urine creatinine were determined using an enzymatic method (Kyowa Medex Co., Ltd, Tokyo, Japan). Urea microalbumin was detected by immunoturbidimetric assay (Beckman Coulter, Inc., CA, USA). The eGFR was calculated according to CKD-EPI under the advice of ADA2021 (23). The following equations were used (24):

**Table:** 

	Scr (mg/dl)	Equation (ml/min per 1.73 m^2^)
Female	≤0.7	144 × (Scr/0.7)^−0.329^ × 0.993^age^
	>0.7	144 × (Scr/0.7)^−1.209^ × 0.993^age^
Male	≤0.9	141 × (Scr/0.9)^−0.411^ × 0.993^age^
	>0.9	141 × (Scr/0.9)^−1.209^ × 0.993^age^

### Definitions

In the study, patients with symptoms of diabetes and either a random blood glucose  ≥11.1 mmol/L, a fasting blood glucose (FBG)  ≥7 mmol/L, or a 2-h blood glucose (BG)  ≥11.1 mmol/L following an oral glucose tolerance test (OGTT) (based on 1999 World Health Organization standards for T2DM), or those who were using glucose-lowering drugs were considered to have T2DM.

DKD was diagnosed based on case history, clinical manifestation, and laboratory examinations. Subjects with UACR ≥300 mg/g and/or eGFR <60 ml/min per 1.73 m^2^ were diagnosed with DKD. Patients with active urinary sediment, rapid progression of albuminuria or nephrotic syndrome, rapidly decreasing eGFR, or the absence of retinopathy were referred to nephrologists for further diagnosis (23).

### Statistical Analysis

Continuous data are presented as mean ± standard deviation (SD) or means with 95% confidence intervals (CIs) and categorical variables as frequencies. Age and DM durations across different groups were assessed using Student’s *t*-test for two groups and ANOVA for three groups. Differences between other continuous variables were assessed by covariance analysis adjusting by age and DM duration. The *χ* ^2^ test was used for difference of gender between groups. Logistic analysis between groups for other categorical variables was adopted, adjusting for age and DM duration and gender. Time-dependent Cox regression model was used to explore the hazard ratios (HRs) and 95% CIs of quartiles of baseline Cysc and latent trajectory classes for incident DKD, with Model 1 remaining unadjusted; Model 2 adjusted for baseline age, gender, and DM duration; and Model 3 adjusted for baseline age, gender, DM duration, smoker, FPG, HbA1c, total cholesterol (TC), triglycerides (TG), urea, uric acid (UA) and Scr. TG and urea were analyzed as time-dependent variables in Model 3. All statistical analyses were performed using the IBM SPSS Statistics 24 software (IBM Corp., Armonk, NY, USA). *p* < 0.05 was considered statistically significant. Time-dependent Cox regression model was conducted using R software (Version 4.0.3, survival package).

Estimations of latent class models were performed using the lcmm package (version 1.9.2) in R (25). LCGMM consists in assuming that the population is heterogeneous and composed of *G* latent classes of subjects characterized by *G* mean profiles of trajectories. Each subject belongs to one and only one latent class. Cysc trajectories were assumed as functions of age in healthy subjects and assumed as a function of DM duration adjusted for age in T2DM subjects. For computation and interpretation purposes, age and DM duration were replaced by age/100 and duration/100. It made the interpretation of the intercepts easier and reduced numerical problems due to very large ages and DM durations in the models. During the model-fitting process, we tested a series of class numbers from 1 to 5, and a series of linear, quadratic, and cubic curves. LCGMM models with 2 or 5 classes were performed several times with a series of random starting values based on the 1-class model. The optimal numbers of classes and curve shapes were determined using Bayesian information criterion (BIC) and mean posterior probabilities as the following criteria: BIC decreased at least 20, high mean posterior class membership probabilities (>0.65), and high mean posterior probabilities (>0.7) (26–28). Finally, according to LCGMM parameters, a 1-class linear model was selected for healthy subjects and a 3-class linear model was selected as the best fit for T2DM subjects, and the final model was described as:


$$Cysc{\left(\text{healthy}\right)}_{\text{ij}}=({\text{υ}}_{0}+{\text{u}}_{0\text{i}})+\left({\text{υ}}_{1}+{\text{u}}_{1\text{i}}\right)\text{duration}+{\text{ϵ}}_{\text{ij}}$$

$$Cysc{\left(\text{DM}\right)}_{\text{ij}}=({\text{υ}}_{0\text{g}}+{\text{u}}_{0\text{ig}})+({\text{υ}}_{1\text{g}}+{\text{u}}_{1\text{ig}})\text{duration}+{\text{ϵ}}_{\text{ij}}$$

where *Cys_ij_* is the outcome value at occasion *j* that is measured at time *t_ij_* of the individual “*i*”, υ = (υ _0g_, υ _1g_) is a vector of fixed-effect parameters in the group “*g*”, *u* = (υ _0_ *_ig_*, *u*_1_ *_ig_*) is a vector of random-effect parameters of the individual “*i*” in the group “*g*”, and ε*_ij_* is an unknown error term.

LCGMM computed fixed-effect parameters (for a class) and random-effect parameters (for an individual). In each latent class, the longitudinal Cysc outcome followed a linear mixed model, including continuous time and intercept in the linear model in the study, with class-specific fixed effects and correlated random effects. Fixed effects represented class-specific mean-predicted parameters. The random effect (Gaussian random deviations) represented the differences between the class-specific fixed effect and the observed values for each individual.

## Results

### Relationship of Serum Cysc With eGFR and UACR

We included 2,924 DM patients between January 1, 2014 and December 31, 2014. At baseline, 1,979 subjects were excluded. Among them, 449 were excluded due to age >70 years, 92 were excluded based on diagnosis of T1DM, 206 were excluded due to UACR ≥30 mg/g, 23 were excluded because of eGFR<60 ml/min per 1.73 m^2^, 1,036 patients were excluded due to previously diagnosed DKD, 2 were excluded due to severe liver dysfunction, 92 were excluded due to malignancy, 73 were excluded because of missing indices, and 6 were excluded based on pregnancy. In the follow-ups, 140 subjects were excluded. Among them, 93 subjects with <3 visits were excluded, 2 were excluded because of pregnancy, 9 were excluded due to malignancy, 4 were excluded because of other nephropathies, and 32 with missing endpoint data were excluded. Finally, 805 subjects were included in the trial, with a mean age of 52.1 ( ± 10.0) years; 459 were men (57.0%).

At the beginning, we computed the latent trajectory of eGFR and UACR by serum Cysc using LCGMM in T2DM subjects, respectively. According to parameters of BIC and mean posterior probabilities, a 1-class quadratic model was selected as the best-fit model for eGFR and a 1-class cubic model for UACR. As shown in **Figure S1**, at 1.1 mg/L of serum Cysc, which is the upper limit of normal reference value (usually, the upper limit of reference is approximately 1.0–1.1 mg/L in different laboratories), the corresponding eGFR and UACR were 98.9 ml/min per 1.73 m^2^ and 16.2 mg/g, respectively. It meant that the increase in serum Cysc was earlier than the clinical diagnosis of DKD by eGFR and UACR. Model parameters are presented in **Tables S1–S4** in the **Supplementary Material**.

### Baseline and Follow-Up Characteristics of Subjects by DKD Incidence

Then, in order to explore the association of Cysc with DKD prevalence, we divided subjects with T2DM into non-DKD and DKD groups by incidence of DKD in the years 2019–2020. **Table 1** presents the baseline characteristics of the DKD and non-DKD groups. Subjects in the DKD group were older and had longer DM duration. In the statistical analysis, covariance analysis was used, adjusted for age and DM duration. At baseline, subjects in the DKD group had higher levels of Cysc, UACR, HbA1c, FPG, TC, TG, urea, and UA, and had a higher proportion of smokers. In the follow-up analysis by incidence of DKD, subjects in the DKD group had a higher Cysc, Scr, body mass index (BMI), DBp, HbA1c, FPG, urea, and UA; a lower eGFR; and a longer DM duration.

**Table 1:** Baseline and follow-up characteristics by incidence of DKD.

	Non-DKD	DKD	*p*
Baseline			
*N* (805)	609	196	
Male [% (*n*)]	55.8 (340)	60.7 (119)	0.23
Age (years)	51.6 (10.0)	53.8 (9.7)	0.005
Duration (years)	6.7 (6.0)	9.4 (6.1)	<0.001
BMI (kg/m^2^)	25.5 (25.2, 25.8)	25.9 (25.3, 26.4)	0.24
SBP (mmHg)	127.6 (126.3, 129.0)	129.6 (127.2, 132.0)	0.167
DBP (mmHg)	81.6 (80.7, 82.5)	83.2 (81.6, 84.8)	0.101
Family history [Yes, % (*n*)]	41.4 (252)	40.3 (79)	0.791
Smoker [Yes, % (*n*)]	17.4 (106)	28.1 (55)	0.001
Drinker [Yes, % (*n*)]	11.0 (67)	13.3 (26)	0.389
HBP [Yes, % *n*)]	36.5 (222)	41.3 (81)	0.979
CHD [Yes, % (*n*)]	11.3 (69)	15.8 (31)	0.995
INFAR [Yes, % (*n*)]	4.6 (28)	4.6 (9)	0.674
UACR (mg/g)	8.7 (8.1, 9.2)	13.6 (12.6, 14.6)	<0.001
HbA1c (%)	8.1 (7.9, 8.2)	9.1 (8.8, 9.4)	<0.001
FPG (mmol/L)	8.8 (8.5, 9.0)	10.0 (9.6, 10.4)	<0.001
TC (mmol/L)	4.76 (4.69, 4.84)	4.94 (4.80, 5.08)	0.037
TG (mmol/L)	2.53 (2.28, 2.77)	3.21 (2.78, 3.63)	0.007
HDL (mmol/L)	1.07 (1.04, 1.09)	1.04 (1.00, 1.08)	0.281
LDL (mmol/L)	2.95 (2.88, 3.02)	2.95 (2.83, 3.07)	0.981
Urea (mmol/L)	5.3 (5.1, 5.4)	5.7 (5.5, 5.9)	0.001
UA (μmol/L)	304.4 (298.1, 310.8)	323.4 (311.8, 336.0)	0.005
Scr (μmol/L)	60.7 (59.6, 61.7)	62.7 (60.8, 64.5)	0.078
CysC (mg/L)	0.90 (0.89, 0.91)	0.94 (0.92, 0.96)	<0.001
eGFR (ml/min per 1.73 m^2^)	105.3 (104.6, 106.0)	104.2 (102.9, 105.4)	0.135
Alb (g/L)	42.7 (42.5, 43.0)	43.6 (43.1, 44.0)	0.004
AST (U/L)	20.0 (18.4, 21.5)	23.3 (20.6, 26.1)	0.04
ALT (U/L)	25.5 (24.2, 26.9)	27.1 (24.7, 29.5)	0.267
Follow-up			
Follow-up time (years)	5.1 (1.4)	4.2 (1.8)	<0.001
Age (years)	56.8 (9.7)	57.2 (10.9)	0.200
Duration (years)	11.8 (6.1)	13.5 (6.3)	0.001
BMI (kg/m^2^)	24.7 (24.4, 25.0)	26.6 (26.0, 27.2)	<0.001
SBP (mmHg)	131.8 (130.0, 133.6)	133.7 (130.3, 137.0)	0.336
DBP (mmHg)	77.9 (76.9, 79.0)	82.5 (80.5, 84.4)	<0.001
UACR (mg/g)	10.5 (8.5, 12.4)	51.9 (48.5, 55.3)	<0.001
HbA1c (%)	7.8 (7.7, 8.0)	8.5 (8.2, 8.7)	<0.001
FPG (mmol/L)	8.0 (7.7, 8.2)	9.3 (8.8, 9.8)	<0.001
TC (mmol/L)	4.60 (4.51, 4.68)	4.65 (4.51, 4.80)	0.507
TG (mmol/L)	2.13 (1.98, 2.28)	2.38 (2.11, 2.65)	0.117
HDL (mmol/L)	1.10 (1.07, 1.12)	1.11 (1.07, 1.16)	0.533
LDL (mmol/L)	2.77 (2.71, 2.83)	2.77 (2.66, 2.88)	0.0945
Urea (mmol/L)	5.18 (5.07, 5.29)	5.66 (5.47, 5.85)	<0.001
UA (μmol/L)	320.0 (312.7, 327.2)	350.1 (337.0, 363.3)	<0.001
Scr (μmol/L)	61.7 (60.6, 62.9)	64.5 (62.4, 66.6)	0.026
CysC (mg/L)	0.90 (0.89, 0.91)	0.95 (0.93, 0.97)	<0.001
eGFR (ml/min per 1.73 m^2^)	100.7 (99.7, 101.7)	99.2 (97.5, 101.0)	0.040
Alb (g/L)	41.6 (41.4, 41.9)	43.4 (42.9, 43.9)	<0.001
AST (U/L)	19.7 (19.1, 20.4)	19.5 (18.3, 20.6)	0.709
ALT (U/L)	24.0 (22.8, 25.2)	22.5 (20.4, 24.6)	0.235

Variables are presented as means (SD), n (%), or means (95% confidence interval) (after covariate analysis).

DKD, diabetic kidney disease; Non-DKD, non-diabetic kidney disease; BMI, body mass index; SBP, systolic blood pressure; DBP, diastolic blood pressure; HBP, high blood pressure; CHD, coronary heart disease; INFAR, cerebral infarction; UACR, urine albumin/creatinine ratio; HbA1c, glycosylated hemoglobin A-1c; FPG, fasting plasma glucose; TC, total cholesterol; TG, triglyceride; HDL, high-density lipoprotein; LDL, low-density lipoprotein; Scr, serum creatinine; UA, uric acid; Cysc, cystatin C; eGFR, estimated glomerular filtration rate; Alb, albumin; AST, aspartate transaminase; ALT, alanine aminotransferase.

eGFR was calculated as the formula for CKD-EPI 2009.

### Hazard Ratios of Quartiles of Baseline Serum Cysc

In the study, the subjects were first designated to 4 quartiles according to baseline Cysc levels. Quartile 1 (Q1) ranged from 0.53 to 0.77 mg/L, Q2 ranged from 0.78 to 1.02 mg/L, Q3 ranged from 1.03 to 1.27 mg/L, and Q4 was greater than 1.28 mg/L. Next, the HRs of the quartiles of Cysc were analyzed for incident DKD. As shown in **Table 2**, in Model 1, the HRs were 1.56 (95% CI, 1.01 to 2.40), 2.35 (95% CI, 1.45 to 3.79), and 9.16 (95% CI, 3.70 to 22.65), respectively, for Q2, Q3, and Q4, when unadjusted. After adjusting for baseline age, gender, and DM duration in Model 2, the HRs were 1.44 (95% CI, 0.93 to 2.24), 1.92 (95% CI, 1.15 to 3.21), and 6.69 (95% CI, 2.65 to 16.87) for Q2, Q3, and Q4, respectively. The Q4 quartile maintained an HR of 7.15 (95% CI, 2.79 to 25.57) after adjusting for baseline age, gender, DM duration, smoker, HbA1c, TC, TG, Urea, UA, and AST in Model 3. The HRs for variables in Model 3 are listed in **Table S5**. The analysis identified Cysc, age, DM duration, HbA1c, UA, aspartate transaminase (AST), DBP, smoker, and urea as risk factors for incidence of DKD.

**Table 2:** Cox regression results of incidence of DKD for quartiles of baseline serum Cysc.

Quartiles of Cysc (mg/L)	Model 1		Model 2		Model 3	
	HR (95% CI)	*p*-value	HR (95% CI)	*p*-value	HR (95% CI)	*p*-value
Q1 (≤0.77)	Reference		Reference		Reference	
Q2 (0.78–1.02)	1.56 (1.01, 2.40)	0.047	1.44 (0.93, 2.24)	0.103	2.05 (1.14, 3.70)	0.017
Q3 (1.03–1.27)	2.35 (1.45, 3.79)	<0.001	1.92 (1.15, 3.21)	0.013	2.30 (1.25, 4.24)	0.008
Q4 (≥1.28)	9.16 (3.70, 22.65)	<0.001	6.69 (2.65, 16.87)	<0.001	7.15 (2.79, 25.57)	<0.001

Model 1, unadjusted by other variables.

Model 2, adjusted for baseline age, gender, and DM duration.

Model 3, adjusted for baseline age, gender, DM duration, smoker, HbA1c, TC, TG, urea, UA and AST. TG and urea are time-dependent covariances.

HR, hazard ratio; 95% CI, 95% confidence interval.

### Trajectory of Cysc in Healthy and T2DM Subjects

**Figure 1:**
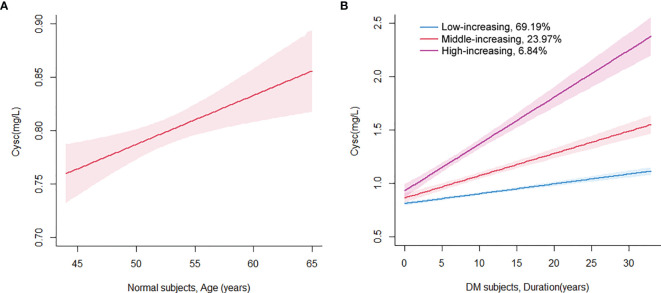
Class-specific mean predicted trajectory of serum cystatin C for healthy subjects **(A)** and T2DM patients **(B)**. A one-class linear model was selected for healthy subjects by age **(A)**, and a three-class linear model was selected for T2DM patients by DM duration as the best-fit models, adjusted for the covariate of age **(B)**.

Healthy subjects from the health checkup database from January 1, 2016 to December 31, 2019 were reviewed. A total of 5,365 subjects had their health checkups for the first time in 2016, and then came back for annual checkups, and were followed for 3 years until 2019. Subjects with diagnosed or new-onset pre-diabetes, DM, hypertension, thyroid disease, malignancy, pregnancy, and other diagnosed diseases and ≤3 visits were excluded. Finally, 349 subjects (142 men, 50.7 ± 3.5 years) were analyzed for trajectory of Cysc. After computing Cysc trajectory by age, testing different classes from 1 to 3, and a series of initial values, according to the parameters of BIC and mean posterior probabilities, a one-class linear model was selected as the best-fit model. As shown in **Figure 1A**, Cysc values increase with age continually. Therefore, in the following analysis, age is considered a confounding variable. Model parameters are presented in **Tables S6, S7** in the **Supplementary Material**. Baseline characteristics of healthy subjects are listed in **Table S8**.

Meanwhile, HCGMM was used to analyze the trajectory of Cysc in DM patients. The trajectory of Cysc was computed by DM durations and adjusted for the covariate of age. After testing different classes from 1 to 5 and different initial values, according to parameters of BIC and mean posterior probabilities, a 3-class linear model was selected as the best-fit model for Cysc trajectory. There were 55 subjects in the high-increasing class, 193 subjects in the middle-increasing class, and 557 subjects in the low-increasing class. As shown in **Figure 1B**, the mean Cysc in the high-increasing class initiated from 0.93 mg/L, with corresponding values of 0.86 mg/L and 0.81 mg/L in the middle- and low-increasing classes, respectively. Cysc in the high-increasing class was increasing at a higher slope at all time points than that in the middle- and low-increasing classes. The slopes of all three classes of DM subjects were higher than that of normal subjects. Model parameters for Cysc in DM subjects are presented in **Tables S9, S10** in the **Supplementary Material**.

### Baseline Characteristics of Latent Classes of Cysc

**Table 3** presents the baseline characteristics of 3 latent classes of Cysc. Subjects in the high-increasing and middle-increasing classes had a higher proportion of male subjects, increased age, and shorter DM duration. Therefore, in the subsequent statistical analysis, we used covariance analysis, adjusted by gender, age, and DM duration. After analysis, at baseline, subjects in the middle-increasing and high-increasing classes had higher BMI, SBp, UACR, HbA1c, FPG, cholesterol, urea, uric acid, and Cysc, and lower eGFR. In follow-up analysis by incidence of DKD, subjects in the middle-increasing and high-increasing classes had a higher proportion of incidence of DKD. The baseline and follow-up characteristics of sub-classes by incidence of DKD are listed in **Table S11**.

**Table 3:** Baseline characteristics by serum Cysc latent classes.

	Low-increasing	Middle-increasing	High-increasing	*p*
Baseline				
*N* (805)	557	193	55	
Male [% (*n*)]	54.8 (305)	58.5 (113)	74.5 (41)	0.016
Age (years)	49.6 (10.0)	58.6 (6.4)	55.2 (8.9)	<0.001
Duration (years)	7.8 (6.6)	6.8 (4.8)	4.0 (3.9)	<0.001
BMI (kg/m^2^)	25.1 (24.8, 25.5)	26.4 (25.8, 27.0)	26.4 (25.3, 27.4)	0.002
SBp (mmHg)	129.7 (128.2, 131.1)	123.9 (121.2, 126.5)	131.1 (126.3, 135.6)	<0.001
DBp (mmHg)	82.5 (81.5, 83.4)	80.5 (78.7, 82.3)	82.7 (79.6, 85.8)	0.15
DM Family history [Yes, % (*n*)]	41.5 (231)	43.0 (83)	30.9 (17)	0.262
Smoker [Yes, % (*n*)]	17.1 (95)	25.9 (50)	29.1 (16)	0.007
Drinker [Yes, % (*n*)]	10.8 (60)	14.0 (27)	10.9 (6)	0.478
HBP [Yes, % (*n*)]	37.9 (211)	35.2 (68)	43.6 (24)	0.514
CHD [Yes, % (*n*)]	12.4 (69)	11.4 (22)	14.5 (9)	0.615
INFAR [Yes, % (*n*)]	4.3 (24)	6.7 (13)	0 (0)	0.092
UACR (mg/g)	9.6 (9.0, 10.3)	11.5 (10.4, 12.6)	7.7 (5.7, 9.7)	0.001
HbA1c (%)	8.1 (8.0, 8.3)	9.0 (8.6, 9.3)	8.3 (7.7, 8.9)	<0.001
FPG (mmol/L)	8.9 (8.6, 9.1)	9.8 (9.4, 10.3)	8.9 (8.1, 9.7)	0.001
TC (mmol/L)	4.74 (4.66, 4.83)	5.12 (4.96, 5.27)	4.78 (4.50, 5.06)	<0.001
TG (mmol/L)	2.51 (2.25, 2.77)	2.90 (2.43, 3.37)	3.31 (2.45, 4.17)	0.15
HDL (mmol/L)	1.09 (1.06, 1.11)	1.06 (1.01, 1.11)	0.97 (0.88, 1.05)	0.031
LDL (mmol/L)	2.93 (2.96, 3.00)	3.16 (3.03, 3.29)	2.76 (2.52, 3.00)	0.001
Urea (mmol/L)	5.1 (5.0, 5.3)	5.8 (5.6, 6.0)	5.4 (5.0, 5.8)	<0.001
UA (μmol/L)	292.2 (285.9, 298.5)	325.0 (313.9, 336.1)	396.8 (375.4, 418.2)	<0.001
Scr (μmol/L)	57.7 (56.9, 58.6)	63.9 (62.4, 65.4)	70.5 (67.8, 73.2)	<0.001
CysC (mg/L)	0.86 (0.85, 0.87)	0.98 (0.97, 1.00)	1.11 (1.08, 1.14)	<0.001
eGFR (ml/min per 1.73 m^2^)	107.0 (106.3, 107.7)	102.0 (100.7, 103.3)	96.4 (94.1, 98.7)	<0.001
Alb (g/L)	42.8 (42.5, 43.1)	43.3 (42.7, 43.8)	42.3 (41.3, 43.2)	0.13
AST (U/L)	19.3 (17.6, 20.9)	24.6 (21.6, 27.6)	26.3 (20.8, 31.7)	0.004
ALT (U/L)	25.0 (23.5, 26.4)	26.0 (23.4, 28.6)	33.5 (28.7, 38.2)	0.004
Follow-up				
Follow-time (years)	4.9 (1.5)	5.0 (1.6)	4.5 (1.9)	0.079
DKD (%, *n*)	21.4 (119)	30.6 (59)	25.5 (14)	0.034

Variables are presented as means (SD), n (%), or means (95% confidence interval) (after covariate analysis).

DKD, diabetic kidney disease; BMI, body mass index; SBp, systolic blood pressure; DBp, diastolic blood pressure; HBP, high blood pressure; CHD, coronary heart disease; INFAR, cerebral infarction; UACR, urine albumin/creatinine ratio; HbA1c, glycosylated hemoglobin A-1c; FPG, fasting plasma glucose; TC, total cholesterol; TG, triglyceride; HDL, high-density lipoprotein; LDL, low-density lipoprotein; Scr, serum creatinine; UA, uric acid; Cysc, cystatin C; eGFR, estimated glomerular filtration rate; Alb, albumin; AST, aspartate transaminase; ALT, alanine aminotransferase.

eGFR was calculated as the formula for CKD-EPI 2009.

### HRs of Latent Classes of Cysc

Finally, we used Cox regression to analyze the HRs for DKD incidence in each latent trajectory class of Cysc. As shown in **Table 4**, in Model 1, the HRs in the high-increasing class and middle-increasing class were 1.45 (95% CI, 0.87 to 2.403) and 1.44 (95% CI, 1.06 to 1.98), respectively, when unadjusted. After adjusting for baseline age, gender, and DM duration in Model 2, the HRs in the high-increasing and low-increasing classes were 2.12 (95% CI, 1.22 to 3.68) and 1.70 (95% CI, 1.18 to 2.44), respectively. In Model 3, the HRs maintained a significance of 3.43 (95% CI, 1.93 to 6.11) for the high-increasing and 1.80 (95% CI, 1.17 to 2.77) for the middle-increasing class after adjusting for baseline age, gender, DM duration, smoker, HbA1c, TC, TG, urea, UA, and AST.

**Table 4:** Cox regression results of incidence of DKD for the latent class of serum Cysc.

	Model 1		Model 2		Model 3	
Latent class	HR (95% CI)	*p*-value	HR (95% CI)	*p*-value	HR (95% CI)	*p*-value
Low-increasing	Reference		Reference		Reference	
Middle-increasing	1.44 (1.06, 1.98)	0.019	1.70 (1.18, 2.44)	0.004	1.80 (1.17, 2.77)	0.007
High-increasing	1.45 (0.87, 2.403)	0.158	2.12 (1.22, 3.68)	0.008	3.43 (1.93, 6.11)	<0.001

Model 1, unadjusted by other variables.

Model 2, adjusted for baseline age, gender, and duration.

Model 3, adjusted for baseline age, gender, DM duration, smoker, HbA1c, TC, TG, urea, UA and AST. TG and urea are time-dependent covariances.

## Discussion

Previous studies have demonstrated that Cysc is an earlier marker than eGFR and UACR associated with a DKD incident. However, a large, longer-duration longitudinal study was needed to further observe the predictive effect of Cysc for DKD. In this study, 805 subjects were included and observed for 5–6 years. The Cysc effect on incidence of DKD was explored from two aspects. At baseline, subjects were segregated into 4 quartiles according to the baseline Cysc values. An HR of 7.15 (95% CI, 2.79 to 25.57) was determined for Q4 when compared with Q1. Subsequently, the Cysc trajectory was analyzed by LCGMM into 3 latent classes. The high-increasing class of Cysc had an HR of 3.43 (95% CI, 1.93 to 6.11) when compared with the low-increasing class. To our knowledge, this is the first large, long-duration longitudinal cohort study on Cysc in DKD, particularly by the latent class analysis of Cysc by LCGMM.

Because of the heavy burden of DKD to patients and society, how to prevent and alleviate DKD is a crucial problem at present. How to discover kidney damage in the early stage is one of the topics. In our study, we computed the dynamic changes of eGFR and UACR with Cysc, and we observed that when the Cysc was already higher than the normal reference, the eGFR and UACR had not met the diagnosis standard of DKD. The following analysis demonstrated that Cysc was associated with the prevalence of DKD. Hence, Cysc is an earlier biomarker than eGFR and UACR associated with future prevalence of DKD. It can help clinical experts to monitor kidney damage in the early stage of DKD to adopt advanced strategies, such as better glucose control, usage of sodium-dependent glucose transporter inhibitors, angiotensin-converting enzyme inhibitors, or administration of uric acid. Cysc offers an alternative to indicate DKD earlier than eGFR and UACR.

In the study, the trajectory of serum Cysc with age was established, and serum Cysc was found to increase with age in normal subjects. There was a slight increase in the velocity of Cysc in normal subjects throughout life (0.46 mg/L per 100 years). This is in accord with previous studies. A study by Norlund et al. found that there were no gender differences for plasma and serum Cysc, whereas an increase in the Cysc levels with age was noted. Reference intervals for serum Cysc in healthy subjects of 0.70–1.21 mg/L for 20–50 years of age and 0.84–1.55 mg/L for over 50 years of age were recommended for practical clinical use (29). An investigation by Finney et al. showed that there were slight differences between genders, so a single reference interval was recommended for each gender. An increase in serum Cysc with age was also observed, and the mean 95% reference interval for those under 50 years of age was 0.53–0.92 mg/L, and for those over 50 years of age, it was 0.58–1.02 mg/L (30). In this study, we analyzed computed Cysc trajectory with age by LCGMM and calculated the velocity of serum Cysc increase by modeling. These findings provide novel insights into the understanding of increasing Cysc with age.

Another important finding in the study is that the increasing velocity of serum Cysc in T2DM subjects with age was faster than that in normal subjects. In T2DM subjects, the slope of Cysc was 0.92, 2.08, and 4.39 mg/L per 100 years for the low-increasing, middle-increasing, and high-increasing classes, respectively. The slopes in all three classes for T2DM subjects were higher than the corresponding slope of 0.46 mg/L per 100 years in normal subjects. In previous studies, some cross-sectional investigations showed that serum Cysc was elevated in DM patients over normal controls (31, 32). However, previous studies did not address the dynamic changes in Cysc in DM patients, especially on the serum Cysc elevation rate, elevation with DM duration, or comparisons with normal controls. To our knowledge, this study is the first investigation on the dynamic changes of Cysc in DM subjects. After analysis, a 2- to 10-fold increasing velocity of serum Cysc was observed in T2DM subjects compared to normal controls. This also indicates a rapid decrease in renal function in DM patients compared to normal subjects.

In the study, subjects were categorized into three latent classes according to serum Cysc increasing velocities. Subjects in the high-increasing class had the highest HR [3.43(95% CI, 1.93 to 6.11)] for incidence of DKD. From the baseline characteristics of the high-increasing class, the data indicate that male subjects with increased age; higher BMI, SBp, urea, UA, Scr, and Cysc; and lower eGFR are more likely to show higher increasing velocity of Cysc. A previous study demonstrated that obesity was associated with increased risk of incidence and progression of DKD (33–35). Weight loss reduced urinary albumin excretion and slowed the decline in GFR (36). Studies on serum UA also demonstrate that higher levels of serum UA are associated with increased risk and progression of DKD in subjects with T1DM and T2DM (37). UA reduction could reduce the rate of GFR loss and decrease the risk of Scr doubling or ESKD in T2DM and other CKD participants (38–40). Combining this and previous studies, attention should be paid to obesity and hyperuricemia, in addition to hyperglycemia and hypertension, to delay DKD incidence in clinical practice.

There are some limitations in our study. Firstly, the endpoint in our study is UACR ≥30 mg/g and/or eGFR<60 ml/min per 1.73 m^2^, so only slight or mild DKD was observed, not severe, especially ESRD. A longer follow-up time is needed. Secondly, because of the influence of SARS-CoV-2, some of the subjects in the study missed visits in 2020. Thus, the observation time was defined as 5–6 years in 2019–2020. However, we adjusted for age and DM duration in the statistical analysis when comparing between groups. Additionally, information on socioeconomic status, occupation, income, education, etc. was absent in our study. Finally, new kidney protective medicines, SGLT-2 inhibitors for example, were not included in the study. A longer observational study, with medicines included, is needed. Nevertheless, because of the large size and long duration of the study, it provides a valuable reference for the study of Cysc in DKD, especially for the latent trajectory of serum Cysc.

In conclusion, this investigation demonstrated that higher baseline Cysc was associated with higher incidence of DKD. DM subjects were divided into 3 latent classes by LCGMM, including low-increasing, middle-increasing, and higher-increasing classes. Subjects in the high-increasing and middle-increasing classes had a higher risk of incidence of DKD. Cysc is a sensitive biomarker for the early diagnosis of DKD.

## Data Availability Statement

The raw data supporting the conclusions of this article will be made available by the authors, without undue reservation.

## Ethics Statement

The studies involving human participants were reviewed and approved by Shengjing Hospital of China Medical University. The patients/participants provided their written informed consent to participate in this study.

## Author Contributions

NW collected data, conceived and designed the experiments, analyzed data, and wrote the manuscript. ZL, WZ and YB collected data. DP and LL conceived and designed the experiments, and revised the manuscript. All authors contributed to the article and approved the submitted version.

## Funding

This work was supported by the Natural Science Foundation of Liaoning Province (No. 2020-MS-149 and No. 2019-ZD-0737), the Science and Technology Talent Program of Shenyang (No. RC200442), and the 345 Talent Project of Shengjing Hospital (No. M0273).

## Conflict of Interest

The authors declare that the research was conducted in the absence of any commercial or financial relationships that could be construed as a potential conflict of interest.

## Publisher’s Note

All claims expressed in this article are solely those of the authors and do not necessarily represent those of their affiliated organizations, or those of the publisher, the editors and the reviewers. Any product that may be evaluated in this article, or claim that may be made by its manufacturer, is not guaranteed or endorsed by the publisher.
