# Supplementary material for: Serum Cystatin C Trajectory Is a Marker Associated With Diabetic Kidney Disease
Source: Front Endocrinol (Lausanne). 2022 May 11;13:824279. doi: 10.3389/fendo.2022.824279 (PMC9130469; doi:10.3389/fendo.2022.824279)
Supplement: Supplementary file 1 [file DataSheet_1.docx]

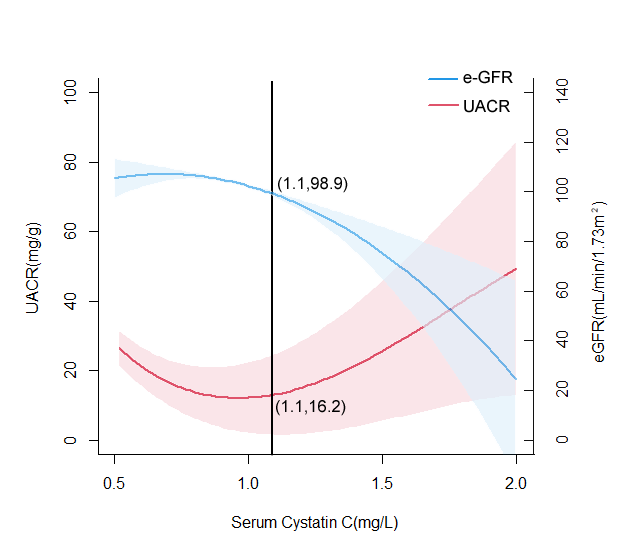


Figure S1. UACR and eGFR changes with serum Cystatin C. Increasing of serum Cystatin C is earlier than clinical diagnosis of diabetic kidney disease by UACR and/or eGFR.

UACR, urinary albumin/creatinine ratio; eGFR, estimated glomerular filtration rate.

Table S1. Model fitting parameters of LCGMM for eGFR by serum Cysc in diabetic subjects.

| No. Latent class | Polynomial degree | Log-lik | npm | BIC | %class1 | %class2 | %class3 | Mean posterior probabilities | % Posterior probabilities>70% |
| --- | --- | --- | --- | --- | --- | --- | --- | --- | --- |
| 1 | Linear | 5734.172 | 6 | -11435.7 | 100 | NA | NA | NA | NA |
|  | **Quadratic** | **5870.554** | **10** | **-11686.7** | **100** | **NA** | **NA** | **NA** | **NA** |
|  | Cubic | 5990.442 | 15 | -11899.2 | 100 | NA | NA | NA | NA |
| 2 | Linear | 5738.04 | 9 | -11427.1 | 3.030303 | 96.9697 | NA | 0.7566/0.9881 | 57.15/99.11 |
|  | Quadratic | 5870.554 | 14 | -11664.9 | 78.35498 | 21.64502 | NA | 0.5117/0.5078 | 0/0 |
|  | Cubic | 5980.895 | 20 | -11852.9 | 19.91342 | 80.08658 | NA | 0.7317/0.7079 | 50.0/43.78 |
| 3 | Linear | 5745.398 | 12 | -11425.5 | 6.060606 | 83.98268 | 9.95671 | 0.8595/0.9463/0.7913 | 85.71/96.39/65.22 |
|  | Quadratic | 5915.482 | 18 | -11733 | 2.164502 | 95.2381 | 2.597403 | 0.8883/0.9909/0.9611 | 80/100/100 |
|  | Cubic | 5989.725 | 25 | -11843.4 | 0 | 100 | 0 | NA/1/NA | NA/100/NA |

Table S2, Fixed effect and random effects parameters of 1 class quadratic LCGMM model for eGFR by serum Cysc in diabetic subjects..

| Fixed effect |  | Coef | Se | P |
| --- | --- | --- | --- | --- |
|  | Intercept | 0.84345 | 0.17712 | <0.001 |
|  | Slope(linear) | 0.66066 | 0.36715 | 0.04195 |
|  | Slope(quadratic) | -0.48044 | 0.19009 | 0.01149 |
| Random effects | | | | |
| σ2 int = 1.48771 |  |  |  | |
| σ2 linear slope = 1.924629 | | |  | |
| σ2 quadratic slope=1 . 0248 | | |  | |
| σ2 error = 0.00044 | |  |  | |

se: standard error

Table S3. Model fitting parameters of Latent Class Growth Mixture Models(LCGMM) for UACR by serum Cysc in diabetic subjects.

| No. Latent class | Polynomial degree | Log-lik | npm | BIC | %class1 | %class2 | %class3 | Mean posterior probabilities | % Posterior probabilities>70% |
| --- | --- | --- | --- | --- | --- | --- | --- | --- | --- |
| 1 | Linear | 355.816595 | 6 | -678.9786838 | 100 | NA | NA | NA | NA |
|  | Quadratic | 498.2566271 | 10 | -942.0890771 | 100 | NA | NA | NA | NA |
|  | **Cubic** | **684.2617959** | **15** | **-1286.887326** | **100** | **NA** | **NA** | **NA** | **NA** |
| 2 | Linear | 355.8165772 | 9 | -662.651395 | 3.03 | 96.97 | NA | 0.5121/0.5078 | 0/0 |
|  | Quadratic | -1.00E+09 | 14 | 2000000076 | 78.35 | 21.65 | NA | 0/NA | 0/NA |
|  | Cubic | -1.00E+09 | 20 | 2000000109 | 19.91 | 80.09 | NA | NA/0 | NA/0 |
| 3 | Linear | 350.9319127 | 12 | -636.5548128 | 6.06 | 83.98 | 9.96 | 0.4078/0.3524/0.3571 | 3.39/0/0 |
|  | Quadratic | -1.00E+09 | 18 | 2000000098 | 2.16 | 95.24 | 2.60 | 0/NA/NA | 0/NA/NA |
|  | Cubic | -1.00E+09 | 25 | 2000000136 | 0 | 100 | 0 | 0/NA/NA | 0/NA/NA |

The best fitting model is highlighted in bold characters. After analysis by different classes and initial values, 1-class cubic model was chosen as the best fitted.

No. Latent class: latent class number of the model; Log-Lik: the maximum Log-Likelihood; BIC: the Bayesian information Criterion; % Participants per class: proportion of participants per class;

NA: not applicable; UACR: urinary albumin/creatinine ratio.

Table S4, Fixed effect and random effects parameters of 1 class quadratic LCGMM model for UACR by serum Cysc in diabetic subjects..

| Fixed effect |  | Coef | Se | P |
| --- | --- | --- | --- | --- |
|  | Intercept | 102.010 | - | - |
|  | Slope(linear) | -207.621 | - | - |
|  | Slope(quadratic) | 144.706 | - | - |
|  | Slope(cubic) | -27.027 | - | - |
| Random effects | | | | |
| σ2 int = 11.42879 |  |  |  | |
| σ2 linear slope = 12.164772 | | |  | |
| σ2 quadratic slope= 12.87056 | | |  | |
| σ2 error = 1.7013 | |  |  | |

se: standard error

Note: the model for UACR by serum Cysc was not convergence criteria satisfied, so the model may need to be computed by larger size in the future. The parameters only reflected the trend of UACR changes by Cysc roughly.

Table S5. Harzard Ratio of baseline variables in Model 3 for incident DKD.

|  | coef | SE(Coef) | | z | Sig. | Exp(B) | | 95% CI for Exp(B) | | | |
| --- | --- | --- | --- | --- | --- | --- | --- | --- | --- | --- | --- |
|  |  |  |  |  |  |  |  | Lower | | Upper | |
| Quartiles of Cysc |  |  | |  |  |  | |  | |  | |
| Q1 | -- | -- | -- | | -- | Reference | -- | | -- | |  |
| Q2 | 0.718 | 0.301 | | 2.385 | 0.017 | 2.050 | | 1.137 | | 3. 698 | |
| Q3 | 0.832 | 0.312 | | 2.671 | 0.008 | 2.300 | | 1.248 | | 4.235 | |
| Q4 | 1.149 | 0.290 | | 3.959 | <0.001 | 7.154 | | 2.786 | | 25.569 | |
| HbA1c(%) | 0.223 | 0.046 | | 4.829 | <0.001 | 1.250 | | 1.142 | | 1.369 | |
| DM duration(years) | 0.065 | 0.014 | | 4.640 | <0.001 | 1.067 | | 1.039 | | 1.097 | |
| Smoker(Yes) | 0.922 | 0.229 | | 4.023 | <0.001 | 2.514 | | 1.604 | | 3.939 | |
| Urea(mmol/L) | 0.229 | 0.068 | | 3.368 | <0.001 | 1.258 | | 1.101 | | 1.437 | |
| AST(U/L) | 0.008 | 0.002 | | 3.272 | 0.001 | 1.008 | | 1.003 | | 1.013 | |
| DBP(mmHg) | 0.026 | 0.009 | | 2.889 | 0.002 | 1.027 | | 1.010 | | 1.044 | |
| UA(μmol/L) | 0.003 | 0.001 | | 2.900 | 0.004 | 1.003 | | 1.001 | | 1.006 | |
| Age | 0.033 | 0.012 | | 2.684 | 0.007 | 1.034 | | 1.009 | | 1.059 | |

HRs of sigificnat variables were shown in this table in Model 3.

DKD, diabetic kidney disease; Cysc, Cystatin C;DM, diabetes mellitus; HbA1c, Glycosylated Hemoglobin A-1c; UA, uric acid; AST, aspartate transaminase;

Quartile 1(Q1) of baseline serum Cysc: ≤0.77 mg/L; Q2: 0.78-1.02 mg/L; Q3: 1.03-1.28 mg/L; and Q4 : ≥0.99mg/L

Table S6, Model fitting parameters of LCGMM for Cysc in healthy subjects.

| No. Latent class | Polynomial degree | Log-lik | npm | BIC | %class1 | %class2 | %class3 | Mean posterior probabilities | % Posterior probabilities>70% |
| --- | --- | --- | --- | --- | --- | --- | --- | --- | --- |
| 1 | **Linear** | **752.2237** | **6** | **-1469.32** | **100** | **NA** | **NA** | **NA** | **NA** |
|  | Quadratic | 752.4937 | 10 | -1446.44 | 100 | NA | NA | NA | NA |
|  | Cubic | 752.9015 | 15 | -1417.98 | 100 | NA | NA | NA | NA |
| 2 | Linear | 760.0227 | 9 | -1467.35 | 91.11748 | 8.882521 | NA | 0.9465/0.7637 | 95.60/58.06 |
|  | Quadratic | 760.3701 | 14 | -1438.77 | 90.83095 | 9.169054 | NA | 0.9421/0.7749 | 95.27/62.50 |
|  | Cubic | 763.0607 | 20 | -1409.02 | 91.11748 | 8.882521 | NA | 0.9428/0.7866 | 96.23/64.52 |
| 3 | Linear | 760.0227 | 12 | -1449.78 | 10.02865 | 89.97135 | 0 | 0.7294/0.6786/NA | 51.43/57.01/NA |
|  | Quadratic | 763.6283 | 18 | -1421.87 | 76.5043 | 1.719198 | 21.7765 | 0.8757/0.8144/0.7462 | 88.39/66.67/60.53 |
|  | Cubic | 752.6934 | 25 | -1359.01 | 0 | 100 | 0 | NA/1/NA | NA/100/NA |

The best fitting model is highlighted in bold characters. After analysis by different classes and initial values, linear 1 class model was chosen as the best fitted.

No. Latent class: latent class number of the model; Log-Lik: the maximum Log-Likelihood; BIC: the Bayesian information Criterion; % Participants per class: proportion of participants per class;

NA: not applicable.

Table S7, Fixed effect and random effects parameters of 1 class linear LCGMM model for Cysc in healthy subjects.

| Fixed effect |  | Coef | Se | P |
| --- | --- | --- | --- | --- |
|  | Intercept | 0.75963 | 0.01396 | <0.001 |
|  | Slope | 0.45905 | 0.14754 | 0.00186 |
| Random effects | | | | |
| σ2 int = 0.01249 |  |  |  | |
| σ2 linear slope = 0.00994 | | |  | |
| σ2 error = 0.00250 | |  |  | |

se: standard error

Table S8, Baseline characteristics of healthy subjects.

| Variables | Mean(SD) |
| --- | --- |
| N | 349 |
| Male(%(n)) | 40.7(142) |
| Age(years) | 50.7(3.5) |
| BMI(kg/m^2^) | 24.4(2.6) |
| SBP(mmHg) | 114.0(10.9) |
| DBP(mmHg) | 71.1(6.7) |
| Smoker(Yes,%(n)) | 16.3(57) |
| Drinker(Yes,%(n)) | 10.9(38) |
| HbA1c(%) | 5.2(0.2) |
| FPG(mmol/L) | 5.2(0.3) |
| TC(mmol/L) | 4.1(0.3) |
| TG(mmol/L) | 1.2(0.8) |
| HDL(mmol/L) | 1.2(0.4) |
| LDL(mmol/L) | 2.5(0.7) |
| UA (μmol/L) | 279.7(74.3) |
| Scr(μmol/L) | 58.6(12.4) |
| CysC(mg/L) | 0.77(0.12) |
| Alb(g/L) | 48.0(3.1) |
| AST(U/L) | 20.6(5.4) |
| ALT(U/L) | 24.5(10.5) |

Data were expressed by Mean(SD) for continuous data, and frequencies for categorical variables. BMI, body mass index; SBP, systolic blood pressure; DBP, diastolic blood pressure; HbA1c, Glycosylated Hemoglobin A-1c; FPG, fasting plasma glucose; TC, total cholesterol; TG, triglyceride; HDL, high density lipoprotein; LDL, low density lipoprotein; UA, uric acid; Scr, serum creatinine; Cysc, Cystatin C; Alb, albumin; AST, aspartate transaminase; ALT, alamine aminotransferase;

Table S9, Model fitting parameters of LCGMM for Cysc in DM subjects.

| No. Latent class | Polynomial degree | loglik | npm | BIC | % Participants per class | Mean posterior probabilities | % Posterior probabilities>70% |
| --- | --- | --- | --- | --- | --- | --- | --- |
| **1** | Linear | -6585.2 | 6 | 13210.6 | 100 | NA | NA |
|  | Quadratic | 2270.306 | 10 | -4473.62 | 100 | NA | NA |
|  | Cubic | 2280.96 | 15 | -4461.43 | 100 | NA | NA |
| 2 | Linear | 2302.826 | 10 | -4538.66 | 11.45/88.55 | 0.7934/0.9515 | 65.59/94.16 |
|  | Quadratic | 2316.32 | 15 | -4532.15 | 10.84/89.16 | 0.8276/0.9572 | 73.86/95.99 |
|  | Cubic | 2338.384 | 21 | -4536.08 | 12.32/87.68 | 0.7884/0.9620 | 65/94.94 |
| **3** | **Linear** | **2320.026** | **14** | **-4566.26** | **69.19/23.97/6.84** | **0.8568/0.7265/0.7620** | **83.60/66.48/68.62** |
|  | Quadratic | 2333.524 | 20 | -4533.06 | 19.58/70.69/9.73 | 0.6491/0.8140/0.7629 | 40.25/74.22/69.62 |
|  | Cubic | 2368.563 | 27 | -4556.24 | 4.31/83.74/11.95 | 0.7983/0.9150/0.7710 | 71.43/92.35/50.52 |
| 4 | Linear | 2333.903 | 18 | -4547.21 | 18.10/7.76/67.24/6.90 | 0.6464/0.6053/0.7972/0.8091 | 36.73/26.98/73.26/75.00 |
|  | Quadratic | 2359.621 | 25 | -4551.75 | 62.81/22.91/7.88/6.40 | 0.8482/0.6864/0.8698/0.7866 | 80.20/39.78/84.38/67.31 |
|  | Cubic | 2358.735 | 33 | -4496.39 | 0/69.70/18.97/11.33 | NA/0.9538/0.9926/0.8165 | NA/94.17/100/73.91 |
| 5 | Linear | 2338.633 | 22 | -4529.88 | 34.36/37.56/8.87/7.02/12.19 | 0.7855/0.7116/0.6059/0.7781/0.5908 | 68.82/54.43/38.89/61.40/27.27 |
|  | Quadratic | 2397.317 | 30 | -4593.65 | 67.86/13.67/4.93/6.65/6.90 | 0.8094/0.6900/0.7596/0.7928/0.7598 | 77.31/41.44/55.0/64.81/55.36 |
|  | Cubic | 2407.771 | 39 | -4554.26 | 6.90/16.50/4.31/16.26/56.03 | 0.7473/0.6824/0.9745/0.9162/0.8237 | 48.21/41.04/100/90.15/75.16 |

The best fitting model is highlighted in bold characters. After analysis by different classes and initial values, linear 3 class model was chosen as the best fitted.

No. Latent class: latent class number of the model; Log-Lik: the maximum Log-Likelihood; BIC: the Bayesian information Criterion; % Participants per class: proportion of participants per class;

NA: not applicable.

Table S10, Fixed effect and random effects parameters of 3 class linear LCGMM model for Cysc in DM subjects.

| Fixed effect |  | Intercept(se)^a^ | Slope(se) |
| --- | --- | --- | --- |
|  | Low-increasing | 0.80904(0.00944) | 0.92093(0.06941) |
|  | Middle-increasing | 0.86281(0.02206) | 2.08427(0.17125) |
|  | High-increasing | 0.93035(0.03171) | 4.38645(0.34336) |
| Random effects | | | |
| σ2 int = 0.00768 |  |  |  |
| σ2 slope = 0.00048 | | |  |
| σ2 error = 0.00141 |  |  |  |

se: standard error

Table S11. Baseline and follow-up characteristics of sub-classes by incident of DKD.

|  | Low-increasing | |  | Middle-increasing | |  | High-increasing | |  |  |
| --- | --- | --- | --- | --- | --- | --- | --- | --- | --- | --- |
|  | Non-DKD | DKD | P | Non-DKD | DKD | P | Non-DKD | DKD | P |  |
| Baseline |  |  |  |  |  |  |  |  |  |  |
| N | 438 | 119 |  | 133 | 60 |  | 38 | 17 |  |  |
| Male(%(n)) | 53.2(233) | 60.5(72) | 0.156 | 59.4(79) | 56.7(34) | 0.721 | 26.3(10) | 23.5(4) | 0.826 |  |
| Age(years) | 49.3(10.0) | 50.5(10.0) | 0.265 | 57.8(6.5) | 60.3(5.6) | 0.011 | 55.5(9.8) | 54.6(6.8) | 0.726 |  |
| Duration(years) | 7.2(6.4) | 10.3(6.5) | <0.001 | 6.0(4.4) | 8.5(5.3) | 0.002 | 3.1(2.6) | 6.1(5.4) | 0.006 |  |
| BMI(kg/m^2^) | 25.4(25.0,25.7) | 25.5(24.7,26.2) | 0.847 | 25.8(25.1,26.6) | 26.0(24.9,27.1) | 0.795 | 26.0(24.6,27.4) | 27.1(25.1,29.1) | 0.401 |  |
| SBP(mmHg) | 128.7(127.1,130.2) | 128.6(125.5,131.7) | 0.971 | 122.7(119.5,126.0) | 131.5(126.8,136.3) | 0.004 | 132.2(126.8,137.6) | 128.9(120.9,136.8) | 0.499 |  |
| DBP(mmHg) | 82.2(81.1,83.3) | 83.4(81.3,85.5) | 0.328 | 79.5(77.6,81.3) | 81.9(79.2,84.6) | 0.151 | 82.0(78.4,85.6) | 86.3(80.9,91.6) | 0.2 |  |
| DM Family history(Yes,%(n)) | 40.6(178) | 44.5(53) | 0.444 | 47.4(63) | 33.3(20) | 0.068 | 28.9(11) | 35.3(6) | 0.638 |  |
| Smoker(Yes,%(n)) | 15.3(67) | 23.5(28) | 0.034 | 27.1(36) | 23.3(14) | 0.584 | 7.9(3) | 76.5(13) | <0.001 |  |
| Drinker(Yes,%(n)) | 11.4(50) | 8.4(10) | 0.347 | 12.8(17) | 16.7(10) | 0.471 | 0.0(0) | 35.3(6) | <0.001 |  |
| HBP(Yes,%(n)) | 36.8(231) | 39.9(87) | 0.534 | 33.8(45) | 38.3(23) | 0.545 | 36.8(14) | 58.8(10) | 0.129 |  |
| CHD(Yes,%(n)) | 12.1(53) | 13.4(16) | 0.693 | 9.8(13) | 15(9) | 0.29 | 7.9(3) | 35.3(6) | 0.032 |  |
| INFAR(Yes,%(n)) | 4.1(18) | 5.0(6) | 0.657 | 7.5(10) | 5.0(3) | 0.737 | 0 | 0 | NA |  |
| UACR(mg/g) | 8.9(8.3,9.5) | 12.8(11.6,14.1) | <0.001 | 8.5(7.3,9.7) | 16.6(14.8,18.4) | <0.001 | 6.3(5.0,7.6) | 9.3(7.5,11.1) | 0.011 |  |
| HbA1c(%) | 8.0(7.8,8.2) | 9.5(9.1,9.9) | <0.001 | 8.7(8.3,9.0) | 8.3(7.8,8.8) | 0.3 | 7.2(6.6,7.8) | 9.5(8.6,10.3) | <0.001 |  |
| FPG(mmol/L) | 8.7(8.4,9.0) | 10.7(10.1,11.3) | <0.001 | 9.3(8.6,9.8) | 8.9(8.2,9.5) | 0.295 | 7.5(6.8,8.3) | 10.2(9.1,11.3) | <0.001 |  |
| TC (mmol/L) | 4.7(4.6,4.8) | 4.9(4.7,5.1) | 0.105 | 4.94(4.75,5.13) | 5.07(4.79,5.36) | 0.449 | 4.5(4.3,4.7) | 5.0(4.7,5.3) | 0.015 |  |
| TG (mmol/L) | 2.6(2.3,2.9) | 3.4(2.8,4.0) | 0.026 | 2.34(1.96,2.72) | 2.51(1.93,3.08) | 0.637 | 2.65(1.76,3.54) | 3.83(2.61,5.05) | 0.132 |  |
| HDL(mmol/L) | 1.08(1.05,1.11) | 1.01(0.95,1.07) | 0.057 | 1.05(1.00,1.10) | 1.14(1.06,1.21) | 0.06 | 0.96(0.92,1.01) | 0.91(0.85,0.96) | 0.11 |  |
| LDL(mmol/L) | 2.90(2.82,2.98) | 2.89(2.74,3.04) | 0.878 | 3.15(2.99,3.32) | 3.17(2.92,3.42) | 0.926 | 2.63(2.40,2.87) | 2.91(2.59,3.23) | 0.183 |  |
| Urea(mmol/L) | 5.1(5.0,5.3) | 5.4(5.2,5.7) | 0.049 | 5.74(5.47,6.01) | 6.05(5.65,6.46) | 0.205 | 5.27(4.85,5.69) | 5.60(5.00,6.21) | 0.384 |  |
| UA(μmol/L) | 299.0(291.9,306.1) | 300.5(286.0,315.0) | 0.856 | 304.4(293.0,315.9) | 333.5(317.1,349.9) | 0.005 | 378.0(335.8,420.3) | 448.5(378.8,518.2) | 0.108 |  |
| Scr(μmol/L) | 0.85(0.84,0.86) | 0.86(0.84,0.88) | 0.285 | 1.00(0.98,1.03) | 1.00(0.96,1.03) | 0.734 | 1.10(1.06,1.14) | 1.15(1.09,1.21) | 0.17 |  |
| CysC(mg/L) | 58.8(57.6,59.9) | 59.5(57.2,61.8) | 0.566 | 64.9(62.8,66.9) | 63.1(60.0,66.3) | 0.38 | 73.0(68.4,77.7) | 72.0(65.0,79.1) | 0.817 |  |
| eGFR( mL/min per 1.73m^2^) | 108.2(107.3,109.1) | 109.7(107.9,111.5) | 0.147 | 97.5(96.2,98.9) | 98.3(96.2,100.3) | 0.555 | 94.9(91.4,98.4) | 95.3(89.9,100.8) | 0.888 |  |
| Alb(g/L) | 42.7(42.4,43.1) | 43.4(42.8,44.0) | 0.062 | 43.0(42.4,43.6) | 43.8(42.9,44.7) | 0.167 | 41.8(40.8,42.8) | 44.1(42.7,45.6) | 0.012 |  |
| AST(U/L) | 19.7(19.0,20.5) | 17.5(16.0,19.0) | 0.009 | 18.5(12.5,24.6) | 35.5(26.4,44.6) | 0.003 | 29.5(25.0,34.0) | 17.2(10.7,23.7) | 0.004 |  |
| ALT(U/L) | 25.7(24.3,27.1) | 24.7(22.0,27.4) | 0.506 | 21.2(17.8,24.6) | 32.7(27.6,37.7) | <0.001 | 39.5(31.4,47.5) | 22.9(11.3,34.5) | 0.025 |  |
| Follow-up |  |  |  |  |  |  |  |  |  |  |
| Follow-time (years) | | 5.1(1.4) | 4.2(1.8) | <0.001 | 5.3(1.4) | 4.4(1.9) | <0.001 |  |  |  |
| Age(years) | 54.3(10.0) | 55.1(9.8) | 0.48 | 63.1(6.7) | 64.5(5.9) | 0.162 | 62.3(7.5) | 53.4(7.9) | <0.001 |  |
| Duration(years) | 12.2(6.5) | 14.5(7.0) | 0.001 | 11.7(4.8) | 12.1(4.6) | 0.567 | 7.8(2.6) | 10.4(4.8) | 0.016 |  |
| BMI(kg/m^2^) | 24.4(24.1,24.8) | 26.8(26.0,27.5) | <0.001 | 26.7(24.1,25.2) | 25.5(24.6,26.4) | 0.122 | 4.9(1.8) | 3.4(1.9) | 0.009 |  |
| SBP(mmHg) | 129.3(127.4,131.2) | 135.7(131.9,139.6) | 0.003 | 139.5(134.6,144.4) | 127.2(119.1,135.2) | 0.011 | 131.9(129.2，134.6) | 141.3(136.4,146.1) | 0.002 |  |
| DBP(mmHg) | 77.9(76.6,79.2) | 82.9(80.4,85.5) | 0.001 | 76.8(74.6,78.9) | 79.7(76.2,83.2) | 0.157 | 83.9(81.1,86.8) | 83.6(78.5.88.7) | 0.917 |  |
| DM Family history(Yes,%(n)) | 10.8(8.5,13.1) | 48.1(43.8,52.4) | <0.001 | 9.9(6.4,13.4) | 59.5(54.1,65.0) | <0.001 | 12.9(4.3,21.5) | 42.0(26.7,57.2) | 0.003 |  |
| Smoker(Yes,%(n)) | 7.8(7.7,8.0) | 8.5(8.2,8.8) | <0.001 | 7.8(7.6,8.1) | 8.5(8.1,8.9) | 0.004 | 7.7(7.2,8.3) | 8.4(7.5,9.3) | 0.24 |  |
| Drinker(Yes,%(n)) | 8.2(7.9,8.6) | 9.8(9.1,10.4) | <0.001 | 7.5(7.1,7.9) | 8.6(8.0,9.3) | 0.005 | 7.2(6.8,7.6) | 7.6(6.9,8.3) | 0.344 |  |
| HBP(Yes,%(n)) | 4.65(4.55,4.75) | 4.75(4.55,4.94) | 0.396 | 4.44(4.29,4.59) | 4.42(4.20,4.65) | 0.924 | 4.59(4.41,4.78) | 4.83(4.49,5.18) | 0.245 |  |
| CHD(Yes,%(n)) | 2.23(2.03,2.43) | 2.44(2.06,2.82) | 0.344 | 1.78(1.59,1.97) | 1.82(1.53,2.11) | 0.81 | 2.37(1.97,2.78) | 3.66(2.90,4.41) | 0.006 |  |
| INFAR(Yes,%(n)) | 1.12(1.09,1.15) | 1.15(1.08,1.21) | 0.448 | 1.06(1.01,1.11) | 1.09(1.02,1.16) | 0.499 | 0.96(0.91,1.02) | 0.94(0.84,1.04) | 0.675 |  |
| UACR(mg/g) | 2.76(2.69,2.84) | 2.79(2.65,2.94) | 0.718 | 2.77(2.65,2.89) | 2.71(2.53,2.89) | 0.565 | 2.82(2.65,2.98) | 2.84(2.54,3.15) | 0.89 |  |
| HbA1c(%) | 5.0(4.9,5.1) | 5.5(5.3,5.8) | <0.001 | 5.60(5.34,5.85) | 5.88(5.50,6.25) | 0.229 | 5.99(5.52,6.46) | 6.15(5.17,7.13) | 0.774 |  |
| FPG(mmol/L) | 313.4(304.9,322.0) | 350.4(333.8,366.9) | <0.001 | 319.1(307.0,331.3) | 339.5(320.1,358.9) | 0.082 | 399.6(357.9,441.3) | 390.4(314.9,465.8) | 0.839 |  |
| TC (mmol/L) | 0.92(0.90,0.94) | 0.96(0.95,0.97) | <0.001 | 1.15(1.12,1.17) | 1.19(1.17,1.20) | 0.009 | 1.41(1.37,1.45) | 1.44(1.36,1.52) | 0.044 |  |
| TG (mmol/L) | 58.8(57.6,60.0) | 59.8(57.5,62.0) | 0.469 | 66.9(64.5,69.3) | 67.5(63.9,71.1) | 0.782 | 76.4(71.4,81.4) | 90.0(80.6,99.4) | 0.019 |  |
| HDL(mmol/L) | 105.7(104.0,107.4) | 104.4(103.5,105.3) | 0.187 | 92.6(90.7,94.6) | 90.2(87.2,93.2) | 0.175 | 87.9(84.1,91.6) | 80.1(73.1,87.1) | 0.069 |  |
| LDL(mmol/L) | 97.2(95.3,99.1) | 93.8(92.8,94.8) | 0.002 | 75.3(73.3,77.3) | 74.6(73.2,75.9) | 0.517 | 65.3(63.1,67.5) | 63.5(59.5,67.5) | 0.039 |  |
| Urea(mmol/L) | 41.9(41.6,42.3) | 43.9(43.3,44.5) | <0.001 | 40.8(40.3,41.3) | 42.0(41.2,42.7) | 0.015 | 41.9(40.8,42.9) | 42.9(41.0,44.9) | 0.373 |  |
| UA(μmol/L) | 19.6(18.9,20.4) | 19.7(18.2,21.2) | 0.952 | 18.5(17.6,19.5) | 19.3(17.9,20.8) | 0.374 | 25.2(20.6,29.9) | 15.9(7.2,24.5) | 0.076 |  |
| Scr(μmol/L) | 25.0(23.5,26.5) | 24.6(21.7,27.4) | 0.798 | 20.1(18.6,21.7) | 19.2(16.9,21.6) | 0.522 | 29.0(23.3,34.8) | 11.8(1.1,22.5) | 0.01 |  |
| Alb(g/L) | 41.9(41.6,42.3) | 43.9(43.3,44.5) | <0.001 | 40.8(40.3,41.3) | 42.0(41.2,42.7) | 0.015 | 41.9(40.8,42.9) | 42.9(41.0,44.9) | 0.373 |  |
| AST(U/L) | 19.6(18.9,20.4) | 19.7(18.2,21.2) | 0.952 | 18.5(17.6,19.5) | 19.3(17.9,20.8) | 0.374 | 25.2(20.6,29.9) | 15.9(7.2,24.5) | 0.076 |  |
| ALT(U/L) | 25.0(23.5,26.5) | 24.6(21.7,27.4) | 0.798 | 20.1(18.6,21.7) | 19.2(16.9,21.6) | 0.522 | 29.0(23.3,34.8) | 11.8(1.1,22.5) | 0.01 |  |

Variables are presented as means (SD) or n (%) or mean(95% confidence interval)(after covariate analysis).

DKD, diabetic kidney disease; Non-DKD, non-diabetic kidney disease; BMI, body mass index; SBp, systolic blood pressure; DBp, diastolic blood pressure; HBP, high blood pressure; CHD, coronary heart disease; INFAR, cerebral infarction; UACR, urine albumin/creatinine ratio; HbA1c, Glycosylated Hemoglobin A-1c; FPG, fasting plasma glucose; TC, total cholesterol; TG, triglyceride; HDL, high density lipoprotein; LDL, low density lipoprotein; Scr, serum creatinine; Cysc, Cystatin C; eGFR, estimated glomerular filtration rate; Alb, albumin; AST, aspartate transaminase; ALT, alamine aminotransferase;

eGFR was calculated as the formula of EPI 2009.
